# Application and recent advances in conventional biomarkers for the prognosis of papillary thyroid carcinoma

**DOI:** 10.3389/fonc.2025.1598934

**Published:** 2025-05-21

**Authors:** Muzi Li, Qiong Li, Cancan Zou, Qin Huang, Yanlin Chen

**Affiliations:** ^1^ Department of Pathology, Women and Children’s Hospital of Chongqing Medical University (Chongqing Health Center for Women and Children), Chongqing, China; ^2^ National Health Commission (NHC) Key Laboratory of Birth Defects and Reproductive Health, Chongqing, China

**Keywords:** papillary thyroid carcinoma, BRAF V600E, TERT, Ras, CD147, tumor microenvironment, ctDNA, microRNA

## Abstract

Cancer remains one of the most common and deadliest diseases worldwide. Among endocrine neoplasms, the incidence of thyroid malignancies has been rising in recent years. Papillary thyroid carcinoma (PTC), the most frequently observed histological subtype of thyroid cancer (THCA), typically yields favorable clinical outcomes for affected individuals. However, this has raised concerns about the overdiagnosis and underdetermination of the prognostic factors in PTC cases. As a result, researchers now advocate for patient stratification and tailored therapeutic approaches for PTC cases, with the goal of minimizing unnecessary surgical procedures and radioiodine treatments. These treatments can lead to clinical complications and impose avoidable physiological and psychological stress on patients. Multiple prognostic biomarkers have been identified for PTC, which play a critical role in predicting outcomes and informing treatment decisions. This review examines both established molecular tools and recent advancements in the determination of prognosis in in papillary thyroid carcinoma.

## 1 Introduction

Among thyroid malignancies, papillary thyroid carcinoma (PTC) is the most common histopathological subtype of thyroid malignancy, accounting for approximately 85-90% of all diagnosed cases (1, 2). Most PTC patients exhibit disease progression similar to benign neoplasms, with minimal mortality, low recurrence rates, and excellent long-term survival outcomes. Limited lymphatic and distant organ involvement further contributes to favorable prognosis observed in many PTC cases (3–5). However, approximately 15-20% (6, 7) of patients experience disease recurrence, persistence, or mortality (8). With PTC incidence steadily rising throughout the past decade, prognostic classification has become increasingly important for therapeutic decision-making, including the evaluation of active surveillance versus surgical management options.

According to the 2022 World Health Organization classification of thyroid neoplasms, multiple histologic subtypes of PTC are recognized, including classic, hypercellular, and follicular varieties. Each subtype may exhibit either encapsulated or infiltrative growth patterns Notably (9), follicular PTC—particularly its encapsulated variant—shares certain pathologic characteristics with follicular thyroid cancer (FTC). Currently, surgical intervention combined with radioiodine therapy remains the primary effective treatment approach for diverse PTC subtypes (10–12). Given the generally favorable outcomes for most PTC patients. Therefore, it is important to distinguish between patients who require active treatment or those who do not. Conventional clinicopathological prognostic indicators have limitations in accurately predicting individual PTC patient outcomes. As a result, a deeper understanding of molecular and transcriptomic profiles may lead to the development of new risk stratification frameworks, facilitating prognostic assessment. This analysis explores both conventional and newly identified biomarkers linked to PTC outcomes, with the aim of developing applicable risk stratification models for integration into clinical practice.

## 2 Conventional biomarkers of PTC prognosis

### 2.1 BRAF V600E mutation in PTC

**Figure 1:**
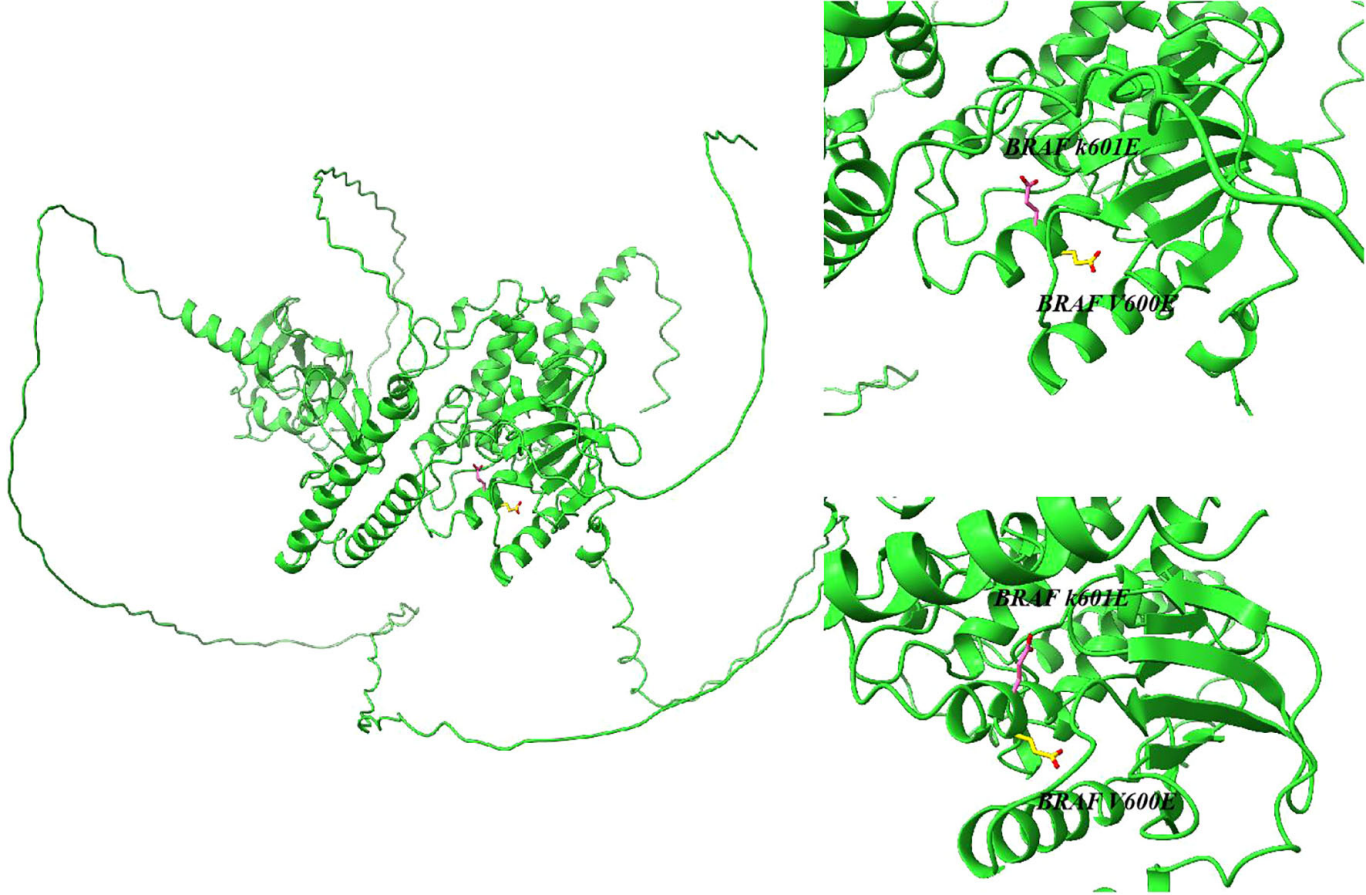
Structure of the serine/threonine kinase encoded by the BRAF K601E and BRAF V600E gene predicted by AlphaFold.

Tumor-associated BRAF gene mutations were first identified in 2002 (13). Subsequently, researchers have gradually characterized over 40 distinct BRAF mutations. Pathologies involving activating BRAF alterations predominantly affect codon 600, yielding V600E (**Figure 1**) mutations, while alternative BRAF-associated conditions exhibit K601E (**Figure 1**) mutations or manifest as minor in-frame insertions, deletions, or structural rearrangements (14, 15). Thyroid malignancies commonly harbor BRAF mutations (16–19). These genomic abnormalities are commonly observed in PTC, poorly differentiated thyroid carcinoma (PDTC), and anaplastic thyroid carcinoma (ATC), while being notably absent in follicular thyroid cancer (FTC), medullary thyroid carcinoma (MTC), and non-malignant thyroid growths.

**Figure 2:**
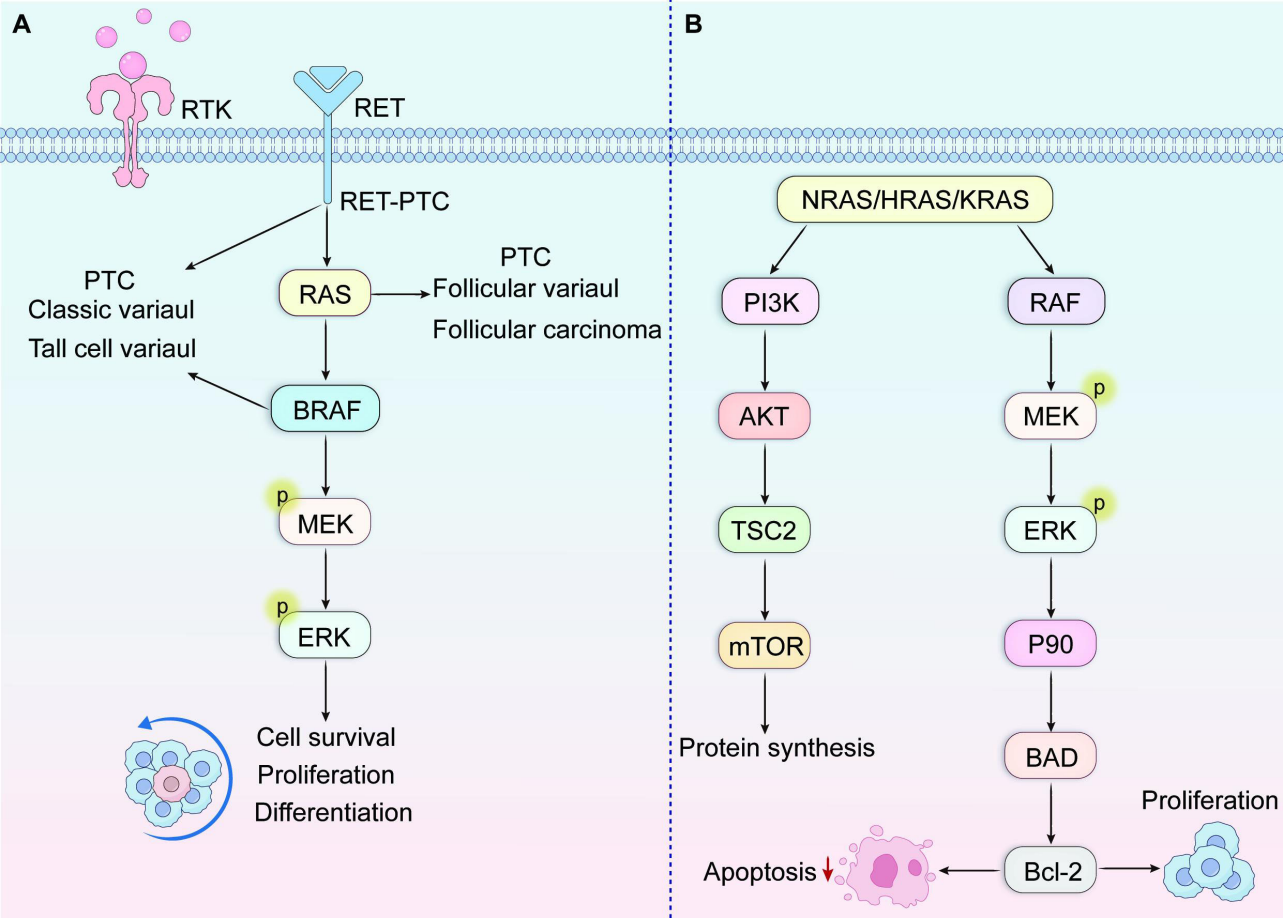
**(A)** MAPK signaling pathway. This pathway binds to growth factors and receptor tyrosine kinase RTK, thereby activating the phosphorylation cascade of RAS, BRAF, MEK, and ERK. **(B)** RAS activation subsequently initiates downstream signaling cascades, predominantly the Raf-MAPK and PI3K-AKT pathways.

Within PTC, BRAF alterations represent the most prevalent genetic alteration, occurring in approximately 36-69% of cases (20, 21). Scientific evidence suggests the BRAF gene encodes a serine/threonine kinase, which acts as an immediate downstream mediator of RAS. Moreover, BRAF promotes continuous kinase activity via MEK and ERK phosphorylation, leading to the tumorigenic activation of the mitogen-activated protein kinase (MAPK) signaling pathway (**Figure 2A**). Somatic activating BRAF mutations are found across various malignancies, notably melanoma (prevalence approaching 70%), and colorectal and ovarian cancers (approximately 15%). According to reports, these mutations promote carcinogenic MAPK pathway activation by disrupting essential interactions that normally preserve the inactive state of the enzyme. Specifically, this genetic alteration interferes with the hydrophobic bonds linking activation loop segments with ATP-binding domain elements, thereby breaking the inactive configuration and creating new molecular interactions that activate the kinase, resulting in persistent enzymatic activity (14, 15). BRAFV600E transfection induces persistent ERK phosphorylation, along with elevated translational activity within NIH3T3 cellular models (22).

### 2.2 TERT

#### 2.2.1 TERT mutations in PTC

As the catalytic component of telomerase responsible for preserving terminal DNA sequences, telomerase reverse transcriptase (TERT) is typically not expressed in most normal human somatic cells, a phenomenon considered crucial for malignancy prevention. In contrast, numerous malignancies exhibit enhanced TERT gene expression through various molecular mechanisms. Predominantly, mutations in the core promoter region of the TERT gene (TERT-p mutations) facilitate cellular immortalization, fundamental characteristic of neoplastic transformation. Cancer-associated TERT-p mutations were first documented in scientific literature during 2013 (23, 24). Comprehensive genomic sequencing examination of melanoma specimens revealed two frequently occurring somatic alterations within the TERT promoter region: mutually exclusive heterozygous cytosine-to-thymine substitutions positioned 124 and 146 base pairs upstream from the translation initiation codon (25). These genetic variants are designated as chr5:1,295,228 C > T (C228T) and chr5:1,295,250 C > T (C250T), respectively, according to human genome reference assembly 19 (hg19) coordinates. Scholars have proposed a biphasic mechanism underlying the contribution of TERT promoter (TERT-p) mutations to oncogenesis (26). Initially, mutations in TERT-p enhance telomerase activity; however, this enhancement only delays cellular aging rather than completely preventing telomere shortening. Subsequently, critically shortened telomeres accumulate, leading to genomic instability, which further elevates TERT expression and ultimately supports cellular proliferative. Indeed, tumors harboring TERT-p mutations exhibit shorter telomeres compared to those in normal tissues (26).

Research has identified TERT-p mutations across various thyroid cancer types, with particularly high frequency in aggressive histological subtypes, such as poorly differentiated thyroid cancer (PDTC) and anaplastic thyroid carcinoma (A TC) (27, 28). These genetic alterations occur across all four principal follicular cell-derived thyroid neoplasms— PTC, FTC, PDTC, and ATC—with predominance in the more aggressive PDTC and ATC variants. Notably, such mutations remain absent in MTC originating from parafollicular C cells. Within PTC specifically, TERT promoter mutations occur in approximately 10% of cases. Recent evidence has established TERT promoter mutations as dependable markers of poor prognosis, supporting their inclusion in clinical management guidelines for thyroid malignancies.

#### 2.2.2 Clinical outcomes and pathological processes in papillary thyroid carcinoma harboring concurrent BRAF V600E and TERT promoter alterations

Extensive studies have established a link between BRAF V600E mutations and increased tumor aggressiveness, including higher rates of recurrence and disease-specific mortality in PTC patients. Similar correlations with adverse clinicopathological parameters have been observed in melanoma (29–32), colorectal neoplasms, and glial tumors. Likewise, TERT promoter mutations are associated with aggressive disease features, such as increased recurrence risk and disease-specific mortality in PTC, as well as greater malignant potential in melanoma, glioma, and urothelial cancers (33). Notably, substantial correlations have been identified between BRAF V600E and TERT promoter genetic changes, with especially frequent simultaneous presence detected in papillary thyroid carcinomas and skin melanomas. While each genetic alteration independently affects cancer prognosis, their simultaneous presence is associated with significantly more aggressive clinical and pathological characteristics. Such features encompass lymph node involvement, remote metastatic spread, higher tumor stages, disease recurrence, and increased mortality specific to PTC among affected individuals. A comprehensive PTC meta-analysis revealed 7.7% prevalence (145/1892 cases) of concurrent BRAF V600E and TERT promoter mutations. Similarly, in melanoma, this mutational co-occurrence correlates with increased tumor dimensions, elevated mitotic indices, lymphatic involvement, ulceration, treatment resistance, heightened recurrence risk, and melanoma-specific mortality (34, 35). Together, this evidence indicates that coexisting BRAF V600E and TERT promoter mutations synergistically drive tumor progression and enhance malignant behavior in papillary thyroid carcinoma.

**Figure 3:**
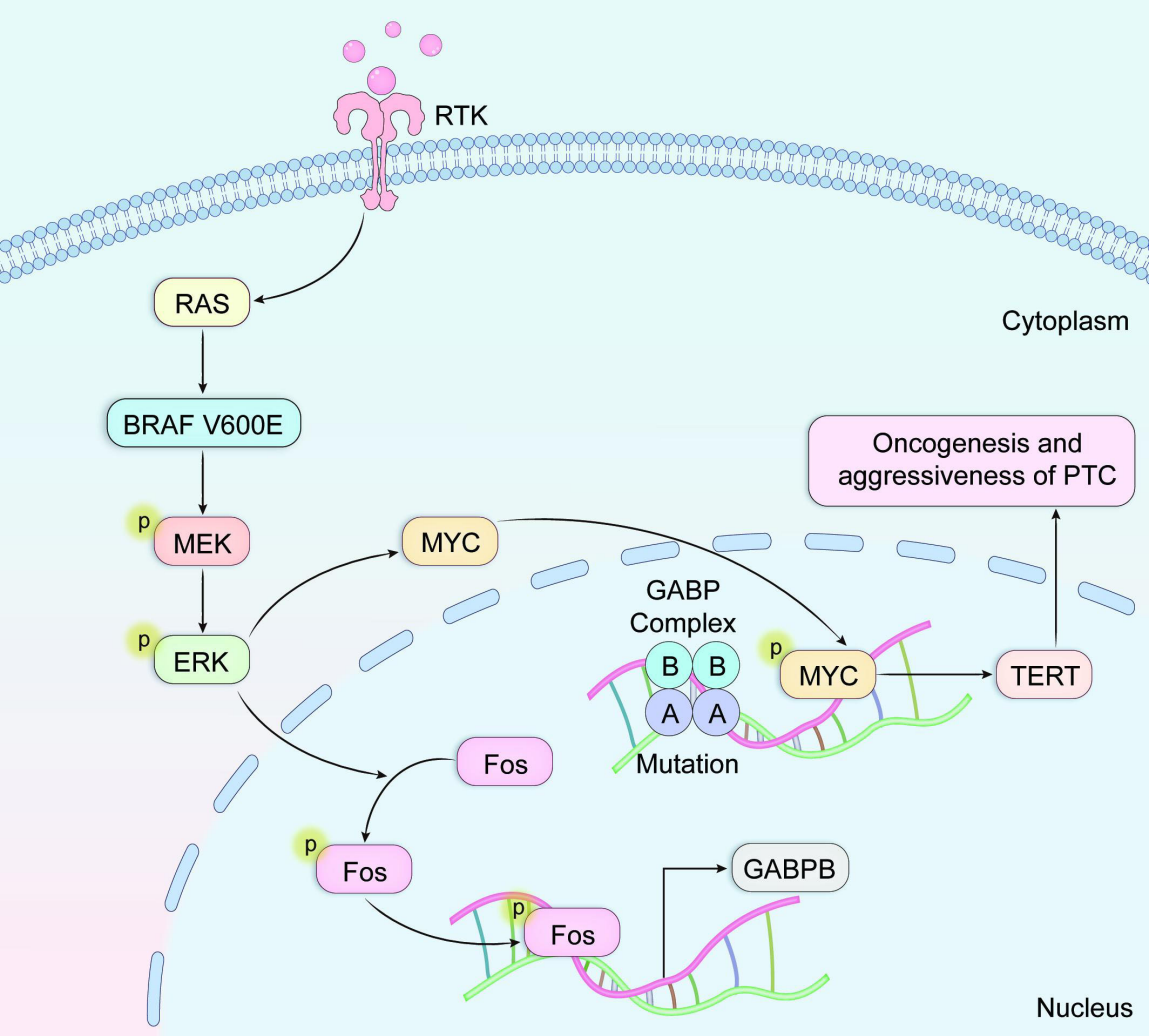
The activated BRAF V600E/MAPK pathway facilitates assembly of transcriptional GABP complexes, subsequently enhancing their recruitment to and transcriptional activation of mutated TERT promoter regions.

Research conducted by Liu and colleagues (36) confirmed a potent synergistic interaction, in which concurrent mutations upregulate TERT expression via the BRAF V600E → MAPK pathway → FOS → GABP → TERT signaling cascade (**Figure 3**). This mechanistic discovery sheds light on the molecular basis underlying the synergistic oncogenic potential observed when BRAF V600E and TERT promoter mutations coexist. The activated BRAF V600E/MAPK pathway facilitates the formation of GABP transcriptional complexes, thereby enhancing their recruitment to and transcriptional activation of mutated TERT promoter regions. Specifically, investigations identified GABPB—the catalytic subunit of the GABP complex, rather than GABPA (the DNA-binding subunit)—as a critical downstream effector within the BRAF V600E/MAPK signaling cascade. effector significantly enhances GABPB transcriptional expression, thereby driving GABP complex formation and subsequently potentiating TERT expression. TERT expression is strongly promoted. Evidence also shows that both MYC and FOS transcription factors interact with the 5’-untranslated region (5’-UTR) of GABPB. Furthermore, BRAF V600E/MAPK signaling promotes FOS phosphorylation, strengthening its binding capacity to GABPB 5′-UTR, which in turn amplifies GABPB expression and activating mutant TERT. FOS-dependent stimulation of GABP transcription via the activated BRAF V600E/MAPK pathways augments mutant TERT promoter activity by promoting the recruitment of conventional RNA polymerase complexes. This mechanism enables mutation-specific regulation of TERT transcription. However, TERT expression can also be regulated independently of TERT promoter status; specifically, BRAF V600E/MAPK signaling can elevate TERT levels through MYC without requiring TERT promoter mutations, even in the absence of TERT promoter mutations, although this effect is comparatively weaker than the promoter-dependent pathway. Consequently, the BRAF V600E/MAPK pathway demonstrates dual regulatory capacity over TERT expression, operating through both mutation-dependent and mutation-independent mechanisms. Within this synergistic model, BRAF V600E mutations primarily enhance cellular proliferative, while TERT promoter mutations mainly contribute to cellular immortalization and survival.

Radioiodine (RAI) therapy constitutes the conventional systemic intervention for differentiated thyroid carcinoma (DTC) presenting with advanced, persistent, recurrent, or metastatic characteristics. Therapeutic responsiveness to RAI administration fundamentally determines clinical outcomes. BRAFV600E mutations demonstrate established correlations with radioiodine refractoriness (RAI-R) through MAPK pathway hyperactivation, which induces cellular dedifferentiation and consequently impairs sodium-iodide symporter (NIS) expression and functionality. Several studies have also confirmed the association of TERT-p mutations with RAI-R (25, 30, 37–40). The clinical significance of this association has been independently validated across diverse geographical cohorts, encompassing both Asian and Western patient populations. Notably, concurrent BRAF V600E and TERT promoter mutations demonstrate significantly stronger predictive capacity for radioiodine refractoriness compared with either mutation occurring independently. Despite relatively limited sample sizes in existing studies, TERT promoter mutations demonstrate remarkably high positive predictive value for therapeutic resistance with the predominant majority of TERT-mutated cases exhibiting radioiodine refractoriness. Furthermore, these genetic alterations demonstrate strong associations with impaired radioiodine uptake capacity (41–44). Conversely, TERT promoter mutation status demonstrates limited sensitivity as a predictive biomarker, evidenced by numerous radioiodine-refractory cases occurring in the absence of these genetic alterations. This observation strongly suggests the existence of alternative molecular mechanisms contributing to therapeutic resistance. Current evidence indicates TERT promoter mutations occur in approximately 24.2-45.5% of radioiodine-refractory cases, representing a significant but incomplete proportion of therapy-resistant patients.

### 2.3 RAS mutations

The RAS proto-oncogene family encompasses three structurally conserved membrane-anchored proteins: NRAS, HRAS, and KRAS, which function as critical molecular switches in signal transduction pathways. Under physiological conditions, RAS activation occurs through guanine nucleotide exchange specifically GDP displacement by GTP facilitated by Grb2/SOS adapter protein complexes. This activation subsequently initiates downstream signaling cascades, predominantly the Raf-MAPK and PI3K-AKT pathways (**Figure 2B**). Such integrated signal transduction systems control multiple vital cellular functions encompassing cell division, specialization, longevity, and metabolic operations (45–47). Specific missense mutations affecting critical codons within RAS genes—particularly codons 12, 13, and 61 in NRAS and HRAS—impair intrinsic GTPase activity necessary for RAS protein inactivation. Consequently, these alterations result in constitutive pathway activation and unregulated downstream signaling. This cancer-promoting process participates in the development of various human tumors, such as pancreatic ductal adenocarcinoma, colorectal cancer, non-small cell pulmonary carcinoma, and differentiated thyroid tumors. Specifically in papillary thyroid carcinoma, RAS gene aberrations appear most frequently within the follicular variant histological pattern.

RAS mutants demonstrate distinctive patterns of downstream pathway activation. Within tumor tissues, research has identified KRAS mutants primarily activating the MAPK pathway, whereas NRAS mutants predominantly amplify PI3K-AKT pathway signaling. Among PTC cases, RAS genomic modifications display reciprocal exclusivity with BRAF mutations (48), indicating functional similarity between RAS and BRAF alterations, while suggesting BRAF mutations can autonomously influence PTC development. Production of aberrant RAS proteins triggers continuous activation of downstream signaling cascades, disturbing cellular equilibrium and fostering abnormal proliferation, compromised differentiation, and heightened survival mechanisms.

Beyond their distinctive function within the PI3K/AKT signaling cascade, RAS gene alterations may simultaneously appear with EIF1AX mutations in patients diagnosed with papillary thyroid carcinoma. In advanced eukaryotic organisms, the process of translation initiation operates under strict control of both cap binding mechanisms and the 43S pre-initiation complexes (PIC). The assembly of PIC requires the ternary complex (EIF2-GTP-tRNAi(Met)) to be incorporated onto the 40S ribosomal subunit. EIF1A, a constituent of PIC, derives from genes located on human chromosomes X and Y, specifically EIF1AX and EIF1AY. According to a previous study (49), protein translation initiation necessitates the complex containing EIF1AX as an essential element. Follicular PTC commonly exhibits isolated alterations within the EIF1AX gene. However, progressive disease stages tend to manifest simultaneous genetic changes affecting both EIF1AX and RAS genes. The literature (25, 37, 50) indicates that C-terminal splice site alterations (A113splice) in EIF1AX appear exclusively in thyroid malignancies, whereas N-terminal structural domain modifications can be detected across various neoplasms, including uveal melanoma. Research investigation 149 examining EIF1AX(A113splice) variants across multiple laboratory and animal models reveals their capacity to substantially elevate mutation frequencies. Experimental systems both outside and within living organisms showed that A113splice variants can collaborate with cancer-causing RAS to accelerate thyroid malignancy development. The prevalent EIF1AX-A113Splice alteration, frequently linked with carcinogenic RAS in thyroid neoplasms, stimulates TF4 production, subsequently triggering widespread protein generation through GADD34-mediated removal of phosphate groups from EIF2α. Additionally, altered EIF1AX, functioning cooperatively with malignancy-driving RAS, enhanced C-MYC protein durability. CMYC and TF4 cooperated to induce transcription of amino acid transporter proteins, and the resulting amino acid flux activated MTOR signaling. These specific mutations may be therapeutically inconvenient (e.g., for MEK, BRD4, and mTOR inhibition).

### 2.4 RET/PTC rearrangement

Initially described by Fusco and colleagues (38), chromosomal rearrangement of RET was first documented in papillary thyroid carcinoma. Positioned at chromosomal locus 10q11.2, the ancestral RET proto-oncogene encodes a transmembrane receptor possessing tyrosine kinase functionality that modulates cellular differentiation and growth mechanisms. Scientific investigations have discovered four separate ligands to date: glial-derived neurotrophic factor (GDN), neurotrophin (NRTN), artemin (ARTN), and persephin (PSPN). These ligands collectively stimulate RET activity via interactions with their respective co-receptor molecules. Expression of RET protein occurs primarily in thyroid par bone or C cells, while scientific consensus regarding its presence in follicular cells of the thyroid continues to be debated.

RET/PTC-associated oncogenic activity emerges through specific chromosomal alterations wherein RET’s C-terminal kinase domain becomes joined with both promoter regions and N-terminal segments from disparate genetic elements (39).

The transformation of the cancer patient’s condition into a genetic disorder linked to the presence of the RET/PTC kinase is triggered by a series of genomic events. These events involve the fusion of the C-terminus of the kinase with the N-terminal sequences of the regulatory elements. In addition, the development of the disorder allows the inappropriate expression of the receptor in the tissue of the thyroid. The fusion of the two components allows the creation of a chimeric oncoprotein that can interact with the SHC articulin adapter proteins. This activity then activates the RAS-RAF-MAPK pathway. According to the scientific literature, there are no fewer than 13 different variants of the RET/PTC rearrangement (40). These genomic alterations exhibit remarkable specificity, appearing predominantly in papillary thyroid carcinoma cases. RET/PTC1 and RET/PTC3 predominate among these genomic alterations, constituting in excess of 90% of documented rearrangement cases (51). Inversions occurring centrally within chromosome 10q generate both RET/PTC1 and RET/PTC3 oncogenes, wherein RET undergoes fusion with activator genes—CCDC6 (alternatively designated H4) and NCOA4 (alternatively termed ELE1 or RFG), respectively. The RET/PTC rearrangement is more frequent in patients less than 45 years of age. Notably elevated frequencies of this rearrangement appear in pediatric papillary thyroid carcinoma patients and individuals with radiation exposure history (47). Research examining pediatric cohorts revealed associations between RET/PTC1 and classical papillary thyroid carcinoma morphology, while solid variant PTC tumors demonstrated greater prevalence of RET/PTC3 rearrangements (39). Micropapillary thyroid carcinomas likewise exhibit substantial RET/PTC rearrangement rates, indicating these genomic alterations likely represent initiating molecular events during papillary thyroid carcinogenesis. Transgenic murine models have established that thyroid-specific expression of RET/PTC1 oncogenes triggers characteristic morphological alterations consistent with PTC development (51). Previously mentioned investigations confirm that detection of RET/PTC rearrangements within RNA isolates from thyroid nodule aspirates provides valuable diagnostic information as one criterion for PTC identification. However, false positive results occasionally occur in certain benign nodular conditions, while accumulated evidence suggests patients harboring RET/PTC-positive PTC predominantly manifest non-aggressive disease without progression to poorly differentiated thyroid malignancies.

### 2.5 Association of traditional biomarkers with clinicopathologic features

Studies have shown that mutations in BRAF and mutations in TERT p are significantly associated with clinicopathologic features of PTC, such as distant metastasis, many metastases in lymph nodes, and advanced development (52, 53). Lee and Tufano et al. conducted a meta-analysis of 26 studies including 1168 and 2470 patients with PTC (54, 55). They reported that BRAF mutations were associated with histologic subtypes, the presence of distant metastases in papillary thyroid carcinoma, and a higher clinical stage, even though the same results were initially published in a meta-analysis by Tufano et al. in Korea. Xing et al. reported the association of BRAF mutations with high recurrence and mortality rates in a large multicenter study, except for the progressive stage and malignant development in patients with PTC, and tiny PTCs (less than 1 cm in diameter) have also been shown to have BRAF mutations, which have been suggested to be an early stage of PTC or a contributing factor to tumorigenesis (56–59). However, in Chen’s study (60), double mutations in BRAF and TERT p were also significantly associated with clinicopathologic features, and as the prevalence of PTC increased, at least 30% of PTC patients had BRAFV600E mutations, of which approximately 10% also had TERTp mutations.The coexistence of BRAFV600E and TERTp mutations strongly predicted a poor prognosis, while The coexistence of BRAFV600E and TERTp mutations strongly predicts poor prognosis, and TERTp is an independent predictor over BRAF. And as older men are more likely to have double mutations, testing for double genes is also recommended for older men.

In Nasirden’s study (61), we found that all patients with TERT p mutations underwent total thyroidectomy, which provides a new perspective on the value of preoperative molecular mutation testing for decision-making on the scope of surgery.TERTp mutations have received widespread attention in recent years, and in addition to their prognostic value, TERTp mutations help determine the significance of surgical scope, prophylactic lymph node dissection, dose of radioiodine therapy, and many other clinical issues, and more high-quality evidence is needed to guide clinical practice. Therefore, the coexistence of the two mutations greatly exacerbates tumorigenesis, metastasis, and lymph node metastasis in PTC, and it has been demonstrated that the two-gene mutations have the following prognostic or therapeutic aggressiveness ranking for PTC patients: BRAF + TERT+ > BRAF - TERT+ > BRAF + TERT- (60).

Despite the overall low mortality rate in PTC patients, our analysis showed that patients with BRAFV600E or TERTp mutations had a poorer prognosis and higher tumor aggressiveness, and that patients carrying dual mutations were significantly associated with recurrence, stage III/IV, and disease-free survival. Patients with double mutations had a 5.85-fold higher risk of aggressiveness and a 31.2-fold higher risk of advanced TNM stage compared to double wild type (62).

RET/PTC1 (CCDC6-RET) and RET/PCT3 (NCOA4-RET) account for 90% of RET rearrangements in patients with PTC. a large cohort study published in 2014 reported mutation rates of 6.8%-10%.RET/PTC mutations are also significantly associated with clinicopathologic features, and they are more likely to occur in younger patients with multifocal lesions and distant metastases (63). Therefore, regardless of the clinical stage, especially when treating patients with recurrent metastases, dual gene testing for RET and BRAF should be performed. Statistical analysis showed that BRAF-positive patients tended to develop lymph node metastasis. In addition, patients with bilateral tumors had a relatively high frequency of BRAF mutations. On the contrary, patients with positive RET status were more likely to be associated with distant metastasis. Moreover, among patients with RET fusion, the number of patients with T4 stage was significantly increased (64).

Whereas RAS mutations are more likely to occur with follicular PTC, studies have shown that RAS is more likely to occur in patients with PTC who have a relatively good prognosis and most of whom have not developed lymph node metastasis and progressed to advanced stages. If preoperative testing of the RAS gene is performed, a better understanding of the extent of lymph node dissection can be obtained (65).

## 3 Recent advances in PTC prognostic biomarkers

### 3.1 CD147 protein levels and genomic instability

CD147 protein,also known as matrix metalloproteinase-inducible protein or extracellular matrix metalloproteinase inducer (EMMPRIN), is a key component of the extracellular matrix metalloproteinase family and plays a crucial role in various cancer progression mechanisms. CD147, a member of the immunoglobulin superfamily, predominantly mediates intercellular interactions and signaling processes, with a structural organization comprising extracellular, transmembrane, and intracellular domains. Functionally, CD147 plays a critical role in regulating tumor biology through multiple mechanisms, including apoptotic pathway modulation via interactions with Bax (66). The interaction of CD147 with various molecules such as integrins and glycoproteins facilitates the modulation of cellular adhesion and migratory capabilities. CD147 activates multiple signal transduction pathways, notably the MAPK/ERK and PI3K/Akt cascades, thereby promoting increased cell proliferation and survival. CD147 expression is elevated in numerous neoplasms, showing a strong correlation with tumor cell invasiveness and metastatic potential. PTC exhibits a significant association between heightened CD147 expression levels and increased tumor aggressiveness, as well as poorer clinical outcomes. There has been considerable scientific interest in the interrelationship between CD147 and chromosomal instability (CIN). CIN, characterized by alterations in chromosome number and structure, is a key feature of tumorigenesis across various malignancies. Studies have shown that CD147 may affect CIN through multiple pathways, including the regulation of cell cycle, DNA damage repair, genome maintenance and other processes.

CIN is a critical feature in the pathogenesis and progression of malignancies, typically presenting as structural and numerical chromosomal aberrations (66, 67). By modulating the expression and function of repair-associated proteins, CD147 disrupts DNA damage repair mechanisms, consequently promoting CIN. Through its modulatory impact on critical checkpoint regulators including p53 and CHK1, CD147 disrupts proper cell cycle advancement and exacerbates CIN. These functions of CD147 are mediated through a large, sometimes overlapping, number of molecular pathways: it transduces signals from upstream molecules or ligands, such as cyclophilin A (CyPA), CD98, and S100A9; activates a range of downstream molecules and pathways, including matrix metalloproteinases (MMPs)-2,3,9, hypoxia-inducible factor (HIF)-1/2a, PI3K/Akt/mTOR/HIF-1a, ATM/ATR/p53; and also regulates monocarboxylate, fatty acid, and amino acid transport proteins (68). And disruption of TP53 has been suggested as an important mechanism to promote CIN propagation *in vitro* and in mouse modeling experiments. Therefore, Elevated CD147 levels potentially augment genomic mutation frequency while additionally promoting CIN through mechanisms involving inflammatory processes and oxidative stress responses. CIN Influence on Recurrence-Free Intervals among PTC Patients. Disease-free survival serves as a key clinical outcome measure, documenting the timespan patients maintain absence of disease recurrence or progression after therapeutic intervention. Scientific studies have demonstrated significant associations between CIN and patient survival trajectories, suggesting that subjects displaying CIN-associated alterations may encounter heightened probability of disease recurrence or progression compared to those without such genetic changes.

**Figure 4:**
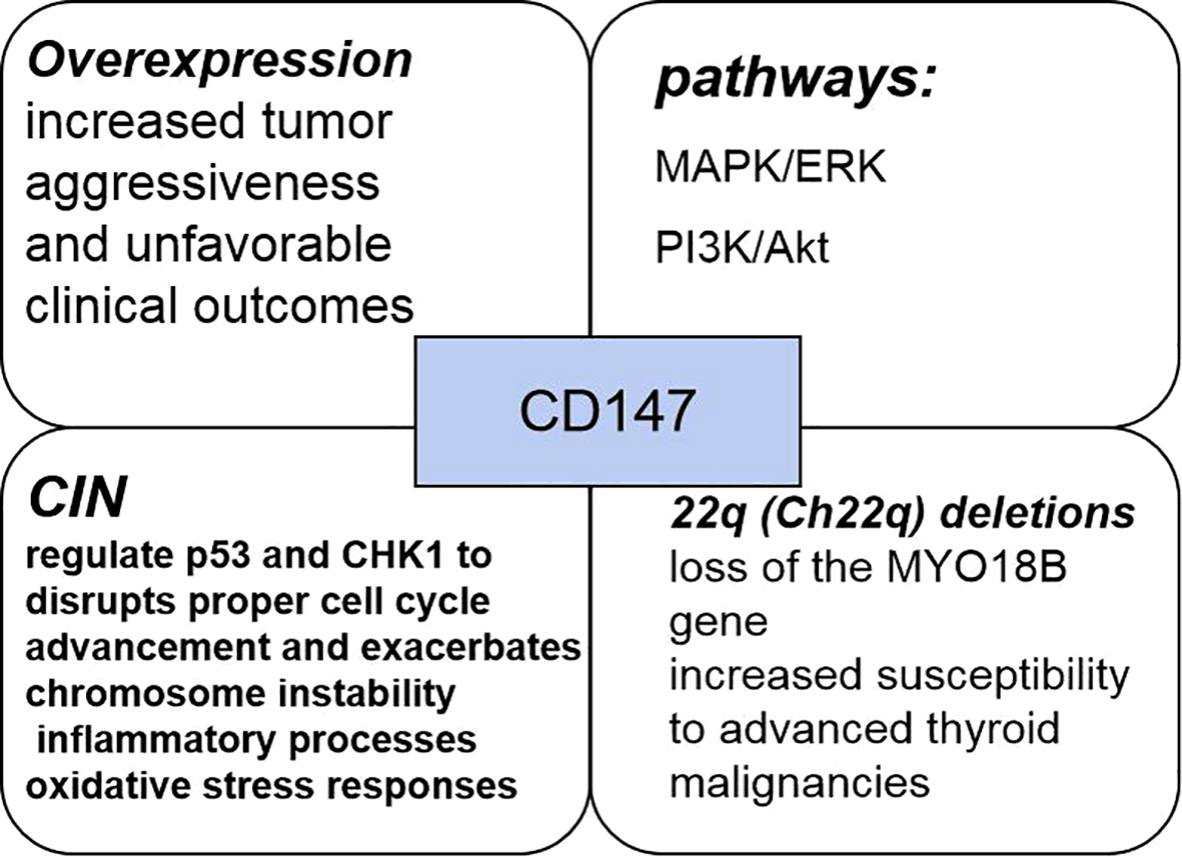
The pathways, mechanisms, and clinical features of CD147 in PTC.

Alterations in genetic material, such as duplications, deletions, rearrangements, and additions, constitute chromosomal abnormalities that can disrupt cellular function, thereby promoting neoplastic transformation (69–71). Previous research has identified 676 genes within chromosome 22q, with specific deletions in this region correlating with developmental disorders, neoplasms, and increased vulnerability to various pathological conditions. The repressor gene MYO18B, situated on the long arm of chromosome 22, is a widely studied genetic variant whose absence appears instrumental in ovarian and colorectal carcinogenesis (72, 73). Patients with PTC frequently exhibit Ch22q deletions and the loss of genetic material from this chromosome’s long arm, which harbors the MYO18B gene, may significantly impact patient outcomes. It has been demonstrated that the incidence of chromosomal abnormalities is higher in intermediate- and high-risk patients, whereas no abnormal chromosomal changes are detected in low-risk patients. Such findings indicate that individuals with chromosomal instability typically exhibit with more aggressive disease characteristics and worse prognostic outcomes compared to those maintaining chromosomal integrity (**Figure 4**). Additionally, patients with chromosomal instability tend to develop larger tumors than those with normal chromosomal profiles. Furthermore, CD147 positivity correlates with increased susceptibility to advanced thyroid malignancies, including hypo-differentiated and undifferentiated variants, while simultaneously predisposing to chromosomal instability—establishing CD147 as a novel prognostic indicator for papillary thyroid carcinoma.

### 3.2 Tumor microenvironment

Comprising malignant cells along with adjacent immune cells, fibroblasts, glial elements, inflammatory mediators, extracellular matrix, and peritumoral vasculature, the tumor microenvironment represents a complex biological ecosystem. Growing evidence suggests that the tumor microenvironment significantly influences neoplastic initiation, growth, invasiveness, and additional pathological mechanisms. Within this complex milieu, immune cellular components exhibit particularly strong associations with cancer progression and clinical outcomes. These elements function as key mediators in immune editing processes, initially providing surveillance and suppressive functions during early carcinogenesis, yet potentially facilitating immune evasion as the malignancy evolves.

Macrophages are the predominant immune cell population within tumor microenvironments and are categorized into three distinct subtypes based on phenotypic and functional characteristics: M0, M1, and M2 (74, 75). M0 macrophages persist in an undifferentiated and dormant condition until exposed to specific polarization stimuli that induce their transformation into functionally specialized M1 or M2 phenotypes. The M1 phenotype exhibits pro-inflammatory properties that enhance immune responses and impede neoplastic advancement, whereas M2 macrophages acts oppositely by facilitating carcinogenesis and malignant progression, thus establishing these distinct macrophage populations as key determinants of tumor behavior. Research indicates that macrophages infiltrating PTC primarily demonstrate M2 polarization (76), playing a significant role in PTC dedifferentiation and facilitating tumor immune evasion processes. 13,23 Additionally, studies have shown that the M0/M2 macrophage infiltration ratios in papillary thyroid carcinoma is significantly higher than those in non-neoplastic thyroid tissue, correlating positively with TNM classification. Conversely, M1 macrophage infiltration is reduced in malignant tissues compared to normal counterparts, exhibiting inverse correlation with TNM parameters. Therefore, it can be concluded that M1 macrophages predominantly infiltrate PTC, the patient is likely to have low-risk PTC, whereas when M0 and M2 macrophages are more abundant, the patient is likely to have high-risk PTC.

Within the neoplastic milieu, lymphocytes represent another critical immune cell population deserving substantial attention. Antigen-specific CD4+ T cells differentiate into specialized CD4+ memory T cell subsets that maintain immunological memory (76–78). Upon secondary antigenic stimulation, CD4+ T cells undergo multiplication and develop into a specific CD4+ T cell subpopulation targeting the pathogen.27,28 Studies have shown that the higher the infiltration ratio of CD4+ memory T cells versus CD8+ T cells in tumor tissues, the lower the risk of PTC. In contrast, a higher ratio of γδ T cell infiltration suggests a higher risk of PTC. Immune cell correlation analysis reveal interactions between macrophages and lymphocytes within the PTC microenvironment. The presence of M1 macrophages is positively associated with infiltration patterns of CD8+ T cells, CD4+ memory T cells, and follicular helper T lymphocytes. Conversely, M2 macrophage abundance demonstrates inverse correlations with these same lymphocyte populations. Therefore, a high percentage of infiltration by M1 macrophages, CD4+ memory T cells, follicular helper T cells, and CD8+ T cells represents low-risk PTC. In contrast, an excessive percentage of infiltration by M0 macrophages, M2 macrophages, and γδ T cells represents high-risk PTC (79).

Through protein-protein interaction network analysis, Li identified pivotal immune-related genes within the neoplastic microenvironment—namely CXCL5, CSF2, ICAM1, CD40LG, and CXCL12—highlighting their associations with both immunological evasion mechanisms and TNM classification parameters (80, 81). Granulocyte-macrophage colony-stimulating factor (CSF2), a hematopoietic growth factor governing myeloid lineage development from precursor cells50, exhibits strong associations with poor clinical outcomes in various cancer types (82). In addition, CSF2 maintains close functional relationships with antigen-presenting cells—specifically dendritic cells and macrophages—within the tumor microenvironment (TME), substantially influencing disease progression and patient outcomes. ICAM1, a transmembrane glycoprotein belonging to the immunoglobulin superfamily, plays essential roles in cellular adhesion, transendothelial leukocyte migration toward inflammatory sites, and lymphocyte activation. These functions consequently modulate neoplastic invasion and metastatic dissemination capacities. The chemokines CXCL5, CCL19, and CCL17 are members of the chemotactic cytokine family closely associated with tumoral angiogenesis. Upon receptor-ligand interaction, these signaling molecules promote malignant progression through various downstream pathways. Existing literature has demonstrated that chemokines CXCL5, CCL19, and CCL17 function as significant mediators in the advancement of diverse malignancies (83, 84). Survival analyses reveal significant prognostic correlations between CD40LG expression and papillary thyroid carcinoma outcomes. Furthermore, bioinformatic interrogation of TCGA dataset indicates potential tumor-suppressive role for this gene in thyroid malignancy. CD40LG ligand (CD40LG), a member of the tumor necrosis factor (TNF) gene superfamily, functions as a cytokine that specifically engages CD40 receptor. This CD40LG-CD40 interaction directly inhibits the proliferation of CD40-expressing neoplastic cells and simultaneously activates immune-mediated tumor suppression through indirect mechanisms (85).

Three immune cell types (M1 macrophages, CD4+ memory T cells, and CD8+ T cells) have been identified to be closely associated with low-risk PTC. In contrast, three immune cell types (M0 macrophages, M2 macrophages, and γδ T cells) and six immune-related genes (CSF2, CXCL5, CCL17, ICAM1, CCL19, and CD40LG) have been identified to be closely associated with high-risk PTC. Based on these associations, it was hypothesized that these cells and genes could be used as biomarkers to predict the prognosis of PTC. Their combined evaluation could help distinguish between high-risk and low-risk subtypes of the disease.

### 3.3 ctDNA and microRNA

ctDNA is derived from DNA fragments found in body fluids including urine, blood and cerebrospinal fluid (86). ctDNA is a very small DNA molecule that can be obtained almost exclusively from tumor cells, and it can be released from tumor cells into the bloodstream through a variety of mechanisms (e.g., apoptosis, necrosis, and secretion) (87, 88). Genetic changes in ctDNA include heterozygosity, mutations, methylation, and copy number alterations (89), which make it a potential biomarker for possible diagnostic treatments. In addition, ctDNA levels are affected by disease severity or disease progression and can vary according to disease progression, site and changes in tumor biology (90). Previous studies have shown that detection of ctDNA is helpful in diagnosing and monitoring cancer patients (91, 92). In addition, the short half-life of ctDNA (less than two hours) makes it possible to monitor tumors in real time. All these findings are sufficient to prove that ctDNA is a potential biomarker for diagnosing tumors and determining their prognosis (93).

Chung et al. (94) found that BRAF mutations could be detected in the circulating DNA of 21% of patients with PTC, whereas mutations in this gene were not present in benign patients. lan et al. (95) found that the detection rate of ctDNA was considerably higher in PTC with distant metastases (DM) than in PTC without DM, and that the rate of detection was correlated with the aggressiveness and the size of the tumor. In addition, ctDNA levels appeared to fluctuate more rapidly with disease status than conventional markers, suggesting that ctDNA may also be one of the biomarkers with therapeutic utility for PTC.

MicroRNA (miRNA) is a class of small RNA molecules that play an important role in the regulation of gene expression in cells. It is a short RNA fragment of about 20 to 22 nucleotides in length. These molecules are crucial in post-transcriptional regulation of gene expression. miRNAs can affect processes such as tumor progression, angiogenesis, invasion and metastasis. Therefore, studying miRNAs in cancer can provide valuable information about tumor biology and possible therapeutic opportunities or biomarkers for cancer diagnosis and monitoring. Several studies have demonstrated the role of miRNA-based analysis in distinguishing high-risk tumor mutations (96)

Some studies have revealed that various miRNAs can serve as biomarkers for the diagnosis of PTC, such as miR-223-3p, miR-34-5p, miR182-5p, miR-146b-5p, miR-29a, miR-223-5p, miR-16-2-3p, miR-34a-5p, miR-346, miR-10a-5p, miR-485-3p, miR-4433a-5p和mir-5189-3p。. In addition, studies have shown that certain miR-31 and miR-21 (e.g., miR-31 and miR-21) can help distinguish between different types of thyroid cancer (e.g., PTC and FTC).MiR-145 has also been identified as a potent marker of increased malignancy in PTC, while miR-6879-5p and miR-6774-3p are diagnostic of lymph node metastasis in PTC, and on the other hand early detection of the presence of recurrence potential in thyroid cancer is crucial. Studies have shown that miRNAs play a role in metastasis and recurrence of PTC. Specific concentrations of exosomal miRNAs (e.g., miR-29a) are associated with recurrence of PTC. In addition, miR-146b and miR-222 can be used as recurrence markers in PTC (93).

Recent studies have evaluated the expression of patient-specific miRNAs in PTC. These miRNAs may serve as useful markers for predicting the likelihood of cancer recurrence after initial treatment. Their early detection in treated patients could help identify those at higher risk of recurrence and allow for more effective and personalized interventions (97).

## 4 Conclusions

**Figure 5:**
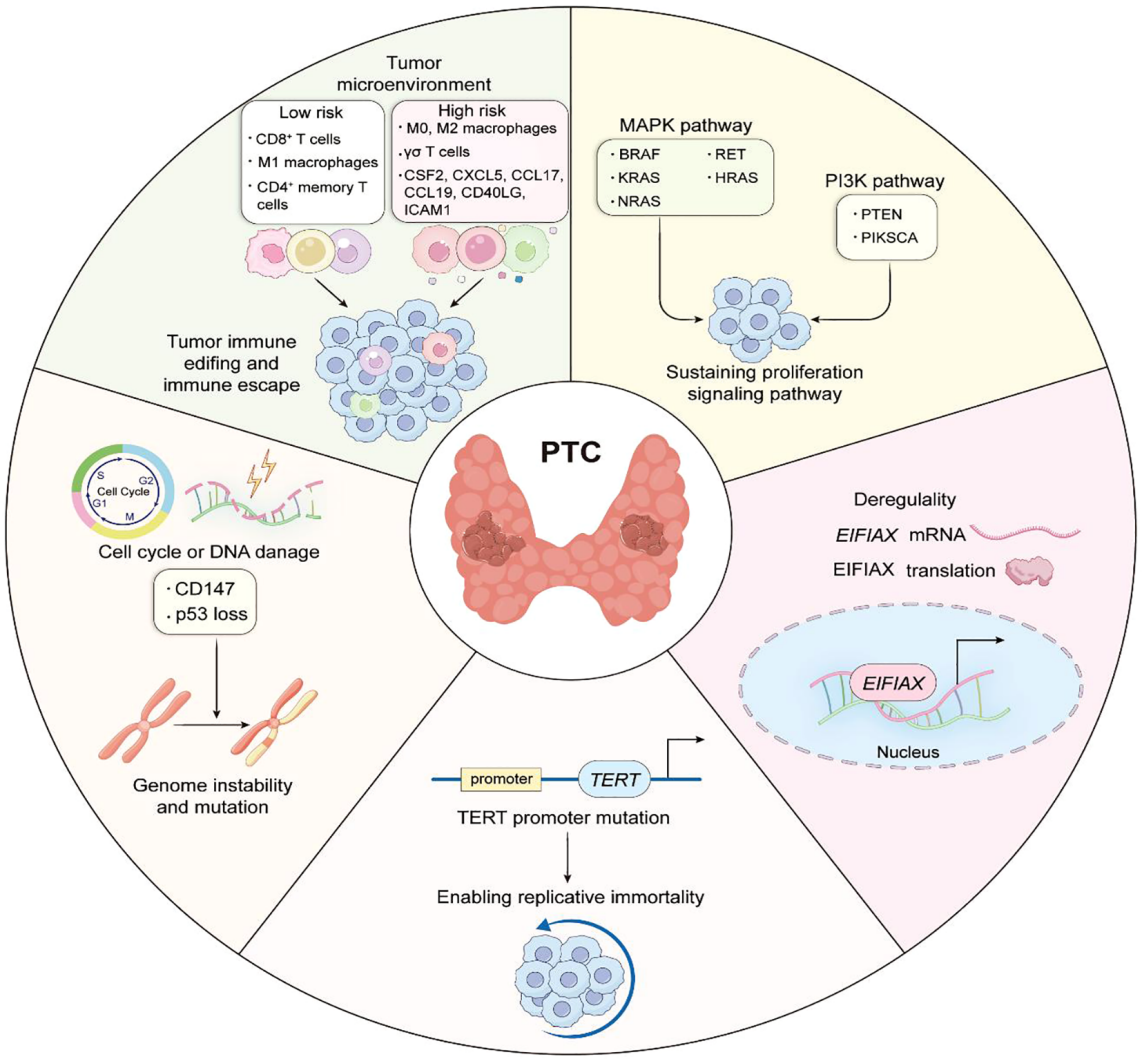
The role of biomarkers in the prognosis of papillary thyroid carcinoma.

Strong advances in molecular research have greatly expanded our understanding of PTC even further and have led to a broader understanding of biomarkers of PTC prognosis and can be used to improve the treatment modalities for patients with PTC through biomarker expression (**Figure 5**, **Table 1**).This review provides traditional as well as novel biomarkers that predict the prognosis of PTC and can be detected by single or multiple genes and cells, for example, mutations in the BRAF and TERT genes imply increased tumor aggressiveness and refractoriness to radioiodine therapy, mutations in the BRAF and RET genes imply an increase in the TMN stage, and mutations in the RAS imply a more favorable The previous traditional biomarkers are already in use in the clinic, and the detection of these genes at the time of thyroid puncture not only provides a basis for an uncertain diagnosis, but also suggests a surgical approach and postoperative treatment. The detection of new biomarkers, including the expression of CD147, which implies increased aggressiveness and chromosomal instability in PTC, and the detection of macrophages and T-cells and their associated immune-expressed genes, which can be used to categorize patients into those with low-risk and high-risk PTC, have not yet been used in the clinic. However, this immunohistochemistry is not specific and has no significance for the diagnosis of PTC, but it has a guiding significance for the potential prognosis of PTC patients, and further experiments and investigations are needed to determine whether there is any correlation with the traditional biomarkers, and the detection of the tumor microenvironment is relatively more complicated, and the current detection of the tumor microenvironment not only consumes more consumables and funds (98), but also leads to inaccurate results, and some studies have shown that the heterogeneity of the tumor can lead to inaccurate results. The tumor heterogeneity can also lead to inaccurate results, and some studies have proved that the tumor microenvironment may be a solution to overcome drug resistance and improve the prognosis of patients (99), but this requires a large number of experiments to prove the research. We know that ctDNA and microRNA are biomarkers with great potential in diagnosing and determining the prognosis of PTC. However, ctDNA and microRNA have very small fragments that are difficult to extract in blood, and it is unstable and easily degraded by various enzymes. Therefore, the analysis of ctDNA and microRNA requires highly sensitive and specific techniques. In recent years, detection strategies for ctDNA and microRNA have become more sophisticated (100). Several techniques are available for analyzing ctDNA, including concentration-based detection and structure-based detection (mutation, methylation). Based on the detection principle, they can be categorized into two types: QPCR and NGS. however, the former can identify only a few known mutations, whereas the latter, although it can identify a large amount of mutation data, must be tested on a large number of samples each time, which makes it difficult to implement within many hospitals.

**Table 1:** Biomarkers associated with prognosis in PTC.

Biomarkers	Signaling Pathway/mechanism	Clinical significance		References
*BRAF V600E*	*BRAF V600E/MAPK*	*High recurrence* *Higher mortality rate* *Worse prognosis*	*BRAF + TERT+ > BRAF − TERT+ > BRAF + TERT−*	(52–59)
*TERT*	*BRAF V600E → MAPK → FOS → GABP → TERT*	*RAI-R* *Poor prognosis* *Increased aggressiveness*		(25, 30, 37–40, 61)
*RAS*	*Raf-MAPK* *PI3K-AKT*	*Follicular PTC* *Good prognosis*		(48, 65)
*RET/PTC*	*RAS-RAF-MAPK*	*Prevalent in younger individuals* *multifocal lesions* *distant metastases*		(40, 63, 64)
*CD147*	*PI3K/Akt/mTOR/HIF-1aATM/ATR/p53*	*CIN* *Poor prognosis* *Larger tumor size*		(68–71)
*ctDNA*	*apoptosis* *necrosis* *secretion*	*BRAF V600E* *Distant Metastasis* *Tumor Size*		(86–90, 93–95)
*microRNA*	*Post transcriptional gene expression regulation*	*Diagnosis of Thyroid Cancer Types* *Recurrence* *High Risk*		(93, 96, 97)
*Tumor Microenvironment*	*Tumor immune editing*	*Distinguish between low-risk and high-risk PTC*		(74–85)

The combined use of biomarkers has been increasingly used in clinical practice, but with the increase in morbidity, it is crucial to choose a more appropriate treatment for patients to achieve better therapeutic efficacy and longer-term survival. Whether it is possible to combine new biomarkers with traditional biomarkers, whether there is a link between them, and how to better utilize them in clinical practice to guide the prognosis of patients are all worthy of further clinical research. deserve further clinical research.
